# Visceral Leishmaniasis Prevalence Forecast for 2040: A Global Overview of 99 Endemic Regions and Countries Using Illness-Death Modeling

**DOI:** 10.34172/jrhs.11503

**Published:** 2026-02-21

**Authors:** Abedin Saghafipour, Meysam Olfatifar, Ehsan Vesali-Monfared, Mahsa Sarvi, Milad Badri, Mahdi Fakhar

**Affiliations:** ^1^Department of Public Health, School of Health, Qom University of Medical Sciences, Qom, Iran; ^2^Gastroenterology and Hepatology Diseases Research Center, Qom University of Medical Sciences, Qom, Iran; ^3^Department of Public Health, School of Health, Hamadan University of Medical Sciences, Hamadan, Iran; ^4^Medical Microbiology Research Center, Qazvin University of Medical Sciences, Qazvin, Iran

**Keywords:** Visceral leishmaniasis, Global burden of disease, Prevalence, Forecasting, Epidemiological models

## Abstract

**Background:**

Visceral leishmaniasis (VL) is a fatal parasitic disease endemic to tropical regions and associated with severe complications and high mortality. Persistent challenges such as delayed diagnosis and limited treatment options highlight the urgent need for robust predictive epidemiological models.

**Study Design:**

This study was a secondary analysis conducted using cross-sectional data.

**Methods:**

Sex-specific VL epidemiological data from 1990 to 2021 were obtained from the Global Burden of Disease (GBD) database and analyzed globally and across 98 endemic countries and regions. An enhanced illness-death model (IDM), incorporating remission, was applied to estimate the age-standardized prevalence rate (ASPR) of VL by 2040.

**Results:**

Globally, the global ASPR of VL decreased by 94.68% between 1990 and 2021. It is projected to decrease by an additional 72.55% by 2040, declining from 0.108 in 2021 to 0.030 (95% CI: 0.020, 0.043) per 100,000 population. In 2040, ASPR is expected to remain higher in males than in females (0.041 vs. 0.019). Tropical Latin America is projected to have the highest regional ASPR at 0.583 (95% CI: 0.565, 0.602). Western Sub-Saharan Africa is the only region expected to experience an increase, rising by 417.12%. Of 83 nations, 27 are expected to exhibit increasing trends, with Djibouti demonstrating the highest projected ASPR at 20.32 (95% CI: 5.25, 78.67), a 2050.59% increase from 2021 to 2040.

**Conclusion:**

Despite global declines in VL prevalence, significant increases are expected in Western Sub-Saharan Africa and countries such as Djibouti, highlighting the need for targeted interventions. Strengthening healthcare systems, improving vector control, and addressing sex-specific risks are crucial to maintain global progress.

## Background

 Visceral leishmaniasis (VL) is a life-threatening parasitic disease that poses a significant global public health challenge, particularly in tropical and subtropical regions where vulnerable and under-resourced communities bear the greatest burden.^1,2^ Transmitted through the bite of some species of infected female phlebotomine sandflies, VL manifests with severe clinical symptoms such as hepatosplenomegaly, pancytopenia, and systemic complications that, if left untreated, result in high mortality.^3^ In many endemic areas, the intersection of poverty, inadequate healthcare infrastructure, and limited access to early diagnostic and treatment services exacerbates disease severity.^1,4^

 Despite substantial advancements in medical technology and therapeutic strategies, persistent barriers remain in the timely diagnosis and effective management of VL.^4^ The disease’s clinical symptoms often resemble those of other febrile illnesses, leading to diagnostic delays that further compromise treatment outcomes.^5^ Moreover, the potential for reinfection since recovery (or remission) does not confer lasting immunity adds another layer of complexity to disease control and prevention efforts.^6^ Compounding these issues, broader environmental and socio-economic factors such as climate change, rapid urbanization, and population displacement are dynamically reshaping the epidemiological landscape of VL.^7^

 Integrated vector management (IVM), a strategic vector control framework promoted by the World Health Organization (WHO) provides five key elements: (1) advocacy, social mobilization, and legislation; (2) collaboration within the health sector and with other sectors; (3) integrated approach to disease control; (4) evidence-based decision-making; and (5) capacity-building.^8-11^ Effective implementation of IVM for sand fly control requires adaptation to changing climatic conditions, primarily through environmental management strategies that modify sand fly habitats to reduce propagation and human-vector contact.^12^ These strategies include environmental modification (long-term physical transformations to reduce larval habitats), environmental manipulation (temporary or seasonal habitat changes), and adjustments to human dwellings or behavior.^10,13,14^

 These factors not only influence the geographic spread of VL but also modify its incidence and mortality patterns, underscoring the urgent need for predictive tools capable of capturing these evolving trends.^4,15^

 In response to these challenges, our study employed advanced epidemiological modeling techniques to forecast the future burden of VL.^16^ Using an enhanced illness-death model (IDM), incorporating remission, we aimed to provide actionable insights to guide public health policies and support targeted interventions in high-burden areas. The IDM framework enables us to offer evidence-based recommendations by capturing the specific factors influencing VL epidemiology at each geographical level and by accounting for temporal fluctuations in epidemiological indices over time. Furthermore, this model considers the interrelationships among these indices, leading to more robust estimates of disease burden. Our analysis is specifically focused on 98 endemic countries/regions, with the global aggregate included as a 99th entity.

## Methods

###  Data collection

 This study utilizes a multi-state modeling framework to forecast the future trajectory of VL up to 2040 at global, regional (15 endemic regions), and 83 national (83 endemic countries) levels. To develop models for each geographical level, we extracted a wide range of VL epidemiological data from the Global Health Data Exchange (GHDx) for the period 1990-2021. The dataset includes sex-specific annual incidence, prevalence, and mortality rates, along with detailed population estimates. By integrating these diverse parameters, our analysis aims to provide a nuanced understanding of VL dynamics, facilitating the development of targeted, evidence-based public health strategies for high-burden areas.

###  Mathematical modeling

 We developed an enhanced IDM framework that categorizes the population into three distinct states: (1) Susceptible, representing individuals at risk of VL infection, (2) Infected, representing individuals diagnosed with VL, and (3) Death, including individuals who die from VL-related complications or other causes. The Global Burden of Disease (GBD) estimates were used as model parameters, except remission, which is not directly available and must therefore be estimated. Transitions from the susceptible to infected state were determined by the annual incidence rate, while transitions from both susceptible and infected states to death were governed by total and cause-specific mortality rates. As mentioned, a key feature of this framework is its ability to model remission, allowing individuals in the infected state to transition back to the susceptible state. Remission was estimated using the following formula^16^:


$$r_{l}=\frac{\left(1-b_{l}^{\left(1\right)}\right)\tau_{k}-\left(1-b_{l}^{\left(0\right)}\right)\tau_{l+1}+\left(1+\tau_{l+1}\right)i_{l}}{\left(1-b_{l}^{\left(1\right)}\right)\tau_{l}}$$

where,

*r_l_* is the remission rate, $b_{l}^{\left(1\right)}$ is the mortality risk for the infected individuals, $b_{l}^{\left(0\right)}$ is the mortality risk for the susceptibles, *τ_l_* is the disease prevalence odds, and i-k is the incidence risk, all at time t. This equation reflects the interrelationship among key epidemiological parameters, enabling the estimation of the remission rate based on mortality, incidence, and prevalence. In other words, this dynamic reflects the clinical reality of VL, where recovery does not guarantee lifelong immunity. In the next step, for each geographical unit and sex, the model was formulated using a system of two discrete-time ordinary differential equations (ODEs), describing transitions among these three epidemiological states while incorporating their interrelationships.


$$\begin{array}{l} S_{t+1}=S_{t}-\rho_{01}S_{t}-\rho_{02}S_{t}+\rho_{10}I_{t}\\ I_{t+1}=I_{t}+\rho_{01}S_{t}-\rho_{12}I_{t}-\rho_{10}I_{t} \end{array}$$

Model calibration was performed using historical GBD data from 1990 to 2021, along with the remission rates estimated in the previous step. Calibration proceeded in two stages: first, a visual inspection of the model fit, followed by the numerical minimization of the root mean square error (RMSE) between model outputs and GBD estimates. This approach ensured that the model accurately captured VL transmission and progression dynamics across different demographic and geographic strata, thereby increasing the reliability of the predictions. Subsequently, the calibrated model was employed to estimate the annual projections of age-standardized prevalence rate (ASPR) for VL through 2040. These projections provide critical insights into the expected burden of the disease in the coming years, enabling public health officials and policymakers to plan and implement targeted interventions effectively. By utilizing robust modeling techniques and historical data, our approach aims to contribute to a more informed understanding of VL’s future trajectory and its implications for global health.

###  Decomposition analysis

 To understand the factors driving changes in disease prevalence and to provide a basis for targeted public health strategies tailored to the specific needs of different regions, we employed the Das Gupta decomposition technique,^17^ which has been widely used in the field of epidemiology for various diseases.^18,19^ This method was selected because it provides an additive meaning the individual contributions of each factor sum precisely to the total observed change and order-independent decomposition ensuring the results are robust and not affected by the sequence in which factors are analyzed.^20^ This analytical method allows us to dissect the contributions of different factors, such as epidemiological dynamics and population growth, to changes in ASPR. In this study, modeling and data analysis were performed using R statistical software (version 4.3.0).

## Results

###  Global prevalence of visceral leishmaniasis 

**Figure 1:**
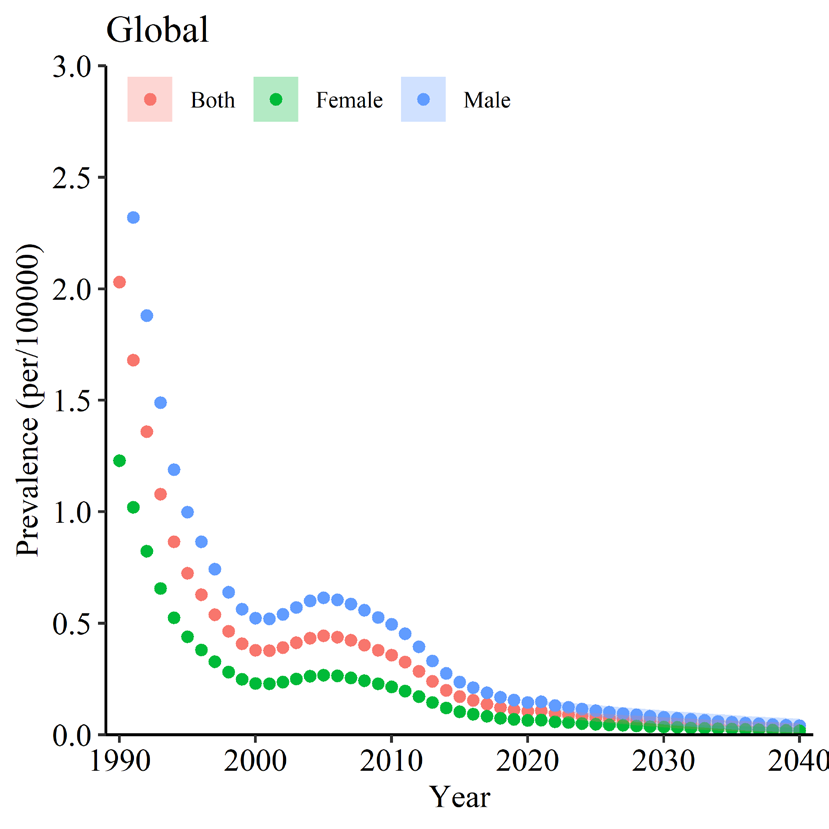
Global observed and projected ASPR of VL from 1990 to 2040. Note. ASPR: Age-standardized prevalence rates; VL: Visceral leishmaniasis. The dotted line represents the observed trend from 1990 to 2021, while the halo effect illustrates the projected trend from 2021 to 2040 with 95% confidence intervals

Figure 1 illustrates the observed and projected global trends in the ASPR of VL from 1990 to 2040. The data indicate a significant decline in the global ASPR, showing a percentage change of -94.68% between 1990 and 2021, with an additional projected decrease of -72.55% from 2021 to 2040. Specifically, the global ASPR is expected to decrease from 0.108 per 100,000 individuals in 2021 to 0.030 (95% CI: 0.020–0.043) per 100,000 by 2040 (Table 1 and Figure 1). Sex-specific analyses demonstrate that females consistently exhibit higher ASPR compared to males. In 2040, the projected ASPR for females is 0.041 (95% CI: 0.028–0.060) per 100,000, while for males, it is 0.019 (95% CI: 0.013–0.028) per 100,000. However, the rate of decrease is slightly less significant in females, with percentage changes from 2021 to 2040 of -71.44% for females and -72.49% for males (Figure 1 and Table 1).

**Table 1:** Global and regional projected ASPR of VL per 100,000 for both sexes in 5-year intervals from 2022 to 2040, including percentage changes from 1990 to 2021 and from 2021 to 2040, as well as RMSE values

**Region**	**ASPR per 100 000 (95% CI)**					**Percentage change**		
	**2022**	**2025**	**2030**	**2035**	**2040**	**1990 vs. 2021**	**2021 vs. 2040**	**RMSE**
Global	0.095 (0.088, 0.102)	0.073 (0.064, 0.084)	0.056 (0.046, 0.069)	0.041 (0.030, 0.055)	0.029 (0.020, 0.043)	-94.679	-72.548	0.020
Central Asia	0.102 (0.098, 0.108)	0.097 (0.090, 0.106)	0.089 (0.078, 0.103)	0.082 (0.067, 0.100)	0.075 (0.058, 0.098)	56.609	-30.417	0.002
Central Europe	0.018 (0.017, 0.019)	0.017 (0.016, 0.019)	0.016 (0.014, 0.018)	0.015 (0.012, 0.018)	0.014 (0.011, 0.018)	-94.394	-32.779	0.007
Southern Latin America	0.003 (0.003, 0.004)	0.002 (0.002, 0.003)	0.001 (0.001, 0.002)	0.001 (0.0005, 0.001)	0.0006 (0.0002, 0.001)	-74.720	-87.522	0.007
Western Europe	0.019 (0.018, 0.019)	0.015 (0.014, 0.016)	0.010 (0.009, 0.011)	0.007 (0.005, 0.008)	0.004 (0.003, 0.006)	-65.884	-77.733	0.007
Andean Latin America	0.010 (0.010, 0.011)	0.009 (0.008, 0.010)	0.007 (0.006, 0.008)	0.005 (0.004, 0.006)	0.004 (0.003, 0.005)	-94.541	-63.498	0.002
Caribbean	0.0005 (0.0005, 0.0005)	0.0004 (0.0004, 0.0005)	0.0004 (0.0003, 0.0004)	0.0003 (0.0003, 0.0004)	0.0003 (0.0002, 0.0004)	-91.537	-40.069	0.004
Central Latin America	0.014 (0.013, 0.014)	0.015 (0.015, 0.016)	0.019 (0.017, 0.021)	0.023 (0.020, 0.026)	0.028 (0.023, 0.033)	-71.551	113.338	0.009
Tropical Latin America	0.640 (0.636, 0.644)	0.630 (0.624, 0.636)	0.614 (0.603, 0.625)	0.598 (0.584, 0.613)	0.583 (0.565, 0.602)	-14.987	-11.347	0.016
North Africa and the Middle East	0.156 (0.145, 0.169)	0.121 (0.106, 0.138)	0.078 (0.063, 0.098)	0.051 (0.037, 0.070)	0.033 (0.022, 0.050)	-92.698	-81.884	0.027
South Asia	0.079 (0.069, 0.091)	0.058 (0.047, 0.073)	0.035 (0.024, 0.052)	0.021 (0.012, 0.036)	0.012 (0.006, 0.026)	-97.979	-86.881	0.081
East Asia	0.0173 (0.016, 0.018)	0.013 (0.012, 0.015)	0.009 (0.008, 0.010)	0.006 (0.005, 0.007)	0.004 (0.003, 0.005)	-81.909	-77.347	0.008
Southeast Asia	0.0002 (0.0002, 0.0002)	0.0002 (0.0002, 0.0002)	0.0001 (0.0001, 0.0002)	0.0001 (0.0001, 0.0002)	0.0001 (8.8e-05-0.0001)	-84.705	-55.911	0.002
Central Sub-Saharan Africa	0.448 (0.361, 0.556)	0.364 (0.253, 0.524)	0.258 (0.139, 0.480)	0.183 (0.076, 0.441)	0.129 (0.041, 0.406)	-94.014	-73.762	0.503
Eastern Sub-Saharan Africa	0.279 (0.257, 0.301)	0.184 (0.161, 0.210)	0.092 (0.073, 0.116)	0.046 (0.033, 0.063)	0.023 (0.015, 0.035)	-97.148	-93.522	0.161
Western Sub-Saharan Africa	0.055 (0.051, 0.060)	0.070 (0.061, 0.080)	0.104 (0.083, 0.131)	0.154 (0.111, 0.213)	0.229 (0.150, 0.348)	269.166	417.116	0.009

*Note.* ASPR: Age-standardized prevalence rates; VL: Visceral leishmaniasis; RMSE: Root mean square error; CI: Confidence interval.

###  Regional prevalence of visceral leishmaniasis 

**Figure 2:**
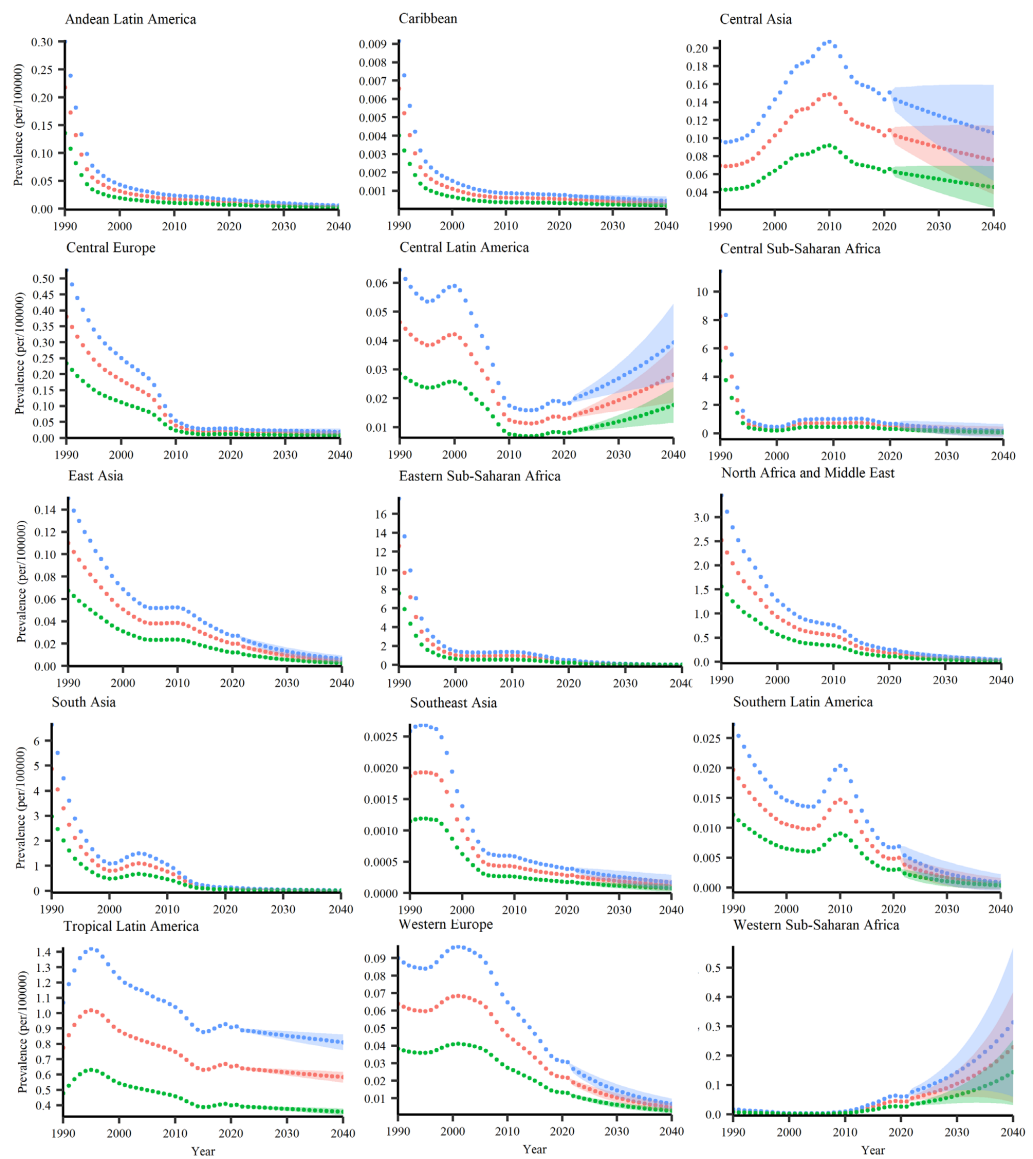
Regional observed and projected ASPR of VL per 100,000 Individuals for Males (blue lines), Females (green lines), and Both Sexes (red lines). Note. ASPR: Age-standardized prevalence rates; VL: Visceral leishmaniasis. The dotted line represents the observed trend from 1990 to 2021, while the halo effect illustrates the projected trend from 2021 to 2040, including 95% confidence intervals

Tables 1-3 and Figure 2 present a comprehensive overview of the projected ASPR of VL and their corresponding percentage changes across 16 regions up to 2040. By 2040, Tropical Latin America stands out with the highest ASPR for both sexes, expected to reach 0.583 (95% CI: 0.565–0.602), despite a modest decrease of -11.35% from 2021 to 2040. Following this, Western Sub-Saharan Africa and Central Sub-Saharan Africa are projected to have the second and third highest ASPRs at 0.229 (95% CI: 0.150–0.348) and 0.129 (95% CI: 0.041–0.406), respectively, with substantial changes of 417.11% and -73.76% from 2021 to 2040 (Figure 2 and Table 1). Similarly, Central Latin America is predicted to experience a 113.34% increase, reaching an ASPR of 0.028 (95% CI: 0.023–0.033) by 2040. Southeast Asia and the Caribbean are forecasted to have the lowest ASPR rates, at 0.00013 and 0.00033, respectively, with decreases of -55.91% and -40.07% from 2021 to 2040. Furthermore, between 1990 and 2021, most regions, except Central Asia and Western Sub-Saharan Africa, exhibited declines in ASPR. Notably, Western Sub-Saharan Africa experienced a significant increase of 269.17%, while Central Asia underwent an increase of 56.61%. However, by 2040, an increasing trend is expected only in Western Sub-Saharan Africa (Table 1 and Figure 2).

**Table 2:** Global and regional projected ASPR of VL per 100,000 females in 5-year intervals from 2022 to 2040, including percentage changes from 1990 to 2021 and from 2021 to 2040, as well as RMSE values

**Region**	**ASPR per 100 000 (95% CI)**					**Percentage change**		
	**2022**	**2025**	**2030**	**2035**	**2040**	**1990 vs. 2021**	**2021 vs. 2040**	**RMSE**
Global	0.058 (0.054, 0.062)	0.048 (0.042, 0.054)	0.035 (0.028, 0.043)	0.025 (0.019, 0.034)	0.018 (0.012, 0.027)	-94.634	-71.437	0.012
Central Asia	0.062 (0.059, 0.065)	0.059 (0.054, 0.064)	0.054 (0.047, 0.062)	0.049 (0.040, 0.061)	0.045 (0.035, 0.059)	52.655	-30.711	0.001
Central Europe	0.011 (0.010, 0.011)	0.010 (0.010, 0.011)	0.010 (0.008, 0.011)	0.009 (0.007, 0.011)	0.008 (0.007, 0.011)	-94.572	-29.666	0.004
Southern Latin America	0.002 (0.002, 0.002)	0.001 (0.001, 0.002)	0.001 (0.0007, 0.001)	0.0006 (0.0003, 0.001)	0.0003 (0.0001, 0.0008)	-74.918	-87.469	0.004
Western Europe	0.011 (0.010, 0.011)	0.009 (0.008, 0.009)	0.006 (0.005, 0.007)	0.004 (0.003-0.005)	0.002 (0.002-0.003)	-65.885	-77.530	0.004
Andean Latin America	0.006 (0.006-0.007)	0.005 (0.005-0.006)	0.004 (0.003-0.005)	0.003 (0.002, 0.004)	0.0026 (0.002, 0.003)	-94.588	-63.327	0.001
Caribbean	0.0003 (0.0003, 0.0003)	0.0002 (0.0002, 0.0003)	0.0002 (0.0002, 0.0003)	0.0002 (0.0001, 0.0002)	0.0002 (0.0001, 0.0002)	-91.542	-39.762	0.003
Central Latin America	0.008 (0.008, 0.009)	0.009 (0.009, 0.010)	0.011 (0.010, 0.013)	0.014 (0.012, 0.016)	0.017 (0.014, 0.021)	-71.678	118.158	0.006
Tropical Latin America	0.392 (0.390, 0.394)	0.386 (0.382, 0.390)	0.376 (0.370, 0.383)	0.366 (0.358, 0.375)	0.357 (0.346, 0.368)	-15.481	-11.488	0.010
North Africa and the Middle East	0.095 (0.088, 0.103)	0.073 (0.064, 0.084)	0.048 (0.038, 0.060)	0.031 (0.022, 0.043)	0.020 (0.013, 0.030)	-92.820	-81.767	0.017
South Asia	0.047 (0.041, 0.054)	0.035 (0.028, 0.044)	0.021 (0.014, 0.031)	0.012 (0.007, 0.022)	0.007 (0.003, 0.015)	-98.010	-86.868	0.048
East Asia	0.010 (0.010, 0.011)	0.008 (0.007, 0.009)	0.005 (0.004, 0.006)	0.003 (0.003, 0.004)	0.002 (0.002, 0.003)	-81.899	-78.170	0.005
Southeast Asia	0.0001 (0.0001, 0.0001)	0.0001 (0.0001, 0.0001)	0.0001 (9.8e-05, 0.0001)	9.7e-05 (7.4e-05, 0.0001)	7.9e-05 (5.5e-05, 0.0001)	-84.347	-56.229	0.001
Central Sub-Saharan Africa	0.277 (0.223, 0.344)	0.225 (0.157, 0.324)	0.159 (0.086, 0.296)	0.113 (0.047, 0.271)	0.080 (0.025, 0.249)	-94.046	-73.804	0.312
Eastern Sub-Saharan Africa	0.170 (0.157-0.184)	0.113 (0.099, 0.129)	0.057 (0.046, 0.072)	0.029 (0.021, 0.040)	0.014 (0.009, 0.022)	-97.131	-93.161	0.101
Western Sub-Saharan Africa	0.034 (0.032-0.037)	0.043 (0.038, 0.049)	0.065 (0.052, 0.080)	0.096 (0.071, 0.131)	0.143 (0.096, 0.213)	262.666	427.817	0.006

*Note.* ASPR: Age-standardized prevalence rates; VL: Visceral leishmaniasis; RMSE: Root mean square error; CI: Confidence interval.

**Table 3:** Global and regional projected ASPR of VL per 100,000 males in 5-year intervals from 2022 to 2040, including percentage changes from 1990 to 2021 and from 2021 to 2040, as well as RMSE values

**Region**	**ASPR per 100 000 (95% CI)**					**Percentage change**		
	**2022**	**2025**	**2030**	**2035**	**2040**	**1990 vs. 2021**	**2021 vs. 2040**	**RMSE**
Global	0.131 (0.122, 0.141)	0.108 (0.095, 0.122)	0.078 (0.063, 0.096)	0.056 (0.042, 0.076)	0.040 (0.027, 0.060)	-94.678	-72.487	0.028
Central Asia	0.142 (0.136, 0.150)	0.136 (0.125, 0.147)	0.125 (0.108, 0.144)	0.115 (0.094, 0.140)	0.105 (0.081, 0.137)	55.991	-29.860	0.004
Central Europe	0.025 (0.024, 0.027)	0.024 (0.022, 0.026)	0.023 (0.020, 0.026)	0.021 (0.017, 0.025)	0.019 (0.015, 0.025)	-94.353	-33.324	0.010
Southern Latin America	0.005 (0.004, 0.006)	0.003 (0.003, 0.005)	0.002 (0.001, 0.003)	0.001 (0.0008, 0.002)	0.0008 (0.0004, 0.001)	-74.652	-87.387	0.009
Western Europe	0.026 (0.025, 0.027)	0.021 (0.019, 0.022)	0.014 (0.012, 0.016)	0.009 (0.008, 0.011)	0.006 (0.005, 0.008)	-66	-77.812	0.001
Andean Latin America	0.015 (0.014, 0.015)	0.012 (0.011, 0.013)	0.009 (0.008, 0.011)	0.007 (0.006, 0.009)	0.005 (0.004, 0.007)	-94.533	-63.638	0.002
Caribbean	0.0007 (0.0006, 0.0007)	0.0006 (0.0006, 0.0007)	0.0005 (0.0005,0.0006)	0.0005 (0.0004, 0.0006)	0.0004 (0.0003, 0.0005)	-91.573	-40.114	0.006
Central Latin America	0.019 (0.019, 0.020)	0.022 (0.021, 0.023)	0.026 (0.024, 0.029)	0.032 (0.028, 0.037)	0.039 (0.032, 0.047)	-71.472	113.649	0.001
Tropical Latin America	0.890 (0.884, 0.895)	0.876 (0.867, 0.885)	0.853 (0.838, 0.869)	0.832 (0.810, 0.853)	0.810 (0.784, 0.838)	-14.485	-11.398	0.022
North Africa and the Middle East	0.215 (0.199, 0.233)	0.166 (0.146, 0.190)	0.108 (0.086, 0.135)	0.070 (0.051, 0.096)	0.045 (0.030, 0.069)	-92.666	-81.853	0.037
South Asia	0.110 (0.096, 0.126)	0.081 (0.064, 0.102)	0.049 (0.033, 0.072)	0.029 (0.017, 0.051)	0.017 (0.008, 0.036)	-97.951	-86.845	0.111
East Asia	0.023 (0.022, 0.024)	0.019 (0.017, 0.020)	0.013 (0.011, 0.015)	0.009 (0.007, 0.011)	0.006 (0.004, 0.008)	-81.933	-76.773	0.001
Southeast Asia	0.0003 (0.0003, 0.0003)	0.0003 (0.0002, 0.0003)	0.0002 (0.0002, 0.0003)	0.0002 (0.0001, 0.0002)	0.0001 (0.0001, 0.0002)	-84.826	-56.095	0.004
Central Sub-Saharan Africa	0.620 (0.499, 0.769)	0.503 (0.350, 0.725)	0.356 (0.191, 0.663)	0.252192 (0.104, 0.608)	0.178 (0.057, 0.558)	-94.011	-73.955	0.697
Eastern Sub-Saharan Africa	0.388 (0.358, 0.420)	0.255 (0.223, 0.291)	0.126 (0.100, 0.159)	0.063 (0.045, 0.087)	0.031 (0.020, 0.047)	-97.154	-93.740	0.220
Western Sub-Saharan Africa	0.077 (0.071, 0.083)	0.097 (0.085, 0.111)	0.144 (0.115, 0.180)	0.212 (0.154, 0.292)	0.314 (0.208, 0.474)	273.493	406.805	0.001

*Note.* ASPR: Age-standardized prevalence rates; VL: Visceral leishmaniasis; RMSE: Root mean square error; CI: Confidence interval.

###  Sex-based regional prevalence of visceral leishmaniasis 

 Among females, by 2040, Tropical Latin America is projected to show the highest ASPR at 0.358, reflecting an -11.40% change from 2021 to 2040. Following this, Western Sub-Saharan Africa is expected to experience an ASPR of 0.144, with a substantial increase of 427.82% from 2021 to 2040 (Table 2 and Figure 2). For males, Tropical Latin America again exhibits the highest ASPR at 0.811, despite a slight decrease of -11.49% from 2021 to 2040. Western Sub-Saharan Africa follows with an ASPR of 0.314, indicating a significant increase of 406.81% from 2021 to 2040 (Table 3 and Figure 2). For both females and males, Southeast Asia and the Caribbean are projected to experience the lowest ASPRs. Notably, analysis of the periods 1990-2021 and 2021-2040 reveals that while most regions experienced declines in ASPR historically, Western Sub-Saharan Africa and Central Latin America were exceptions during 1990-2021. In contrast, from 2021 to 2040, only Western Sub-Saharan Africa is expected to exhibit a substantial increase in ASPR (Tables 2-3 and Figure 2). These results suggest a consistently higher ASPR of VL among males compared to females.

###  National prevalence of visceral leishmaniasis 

**Figure 3:**
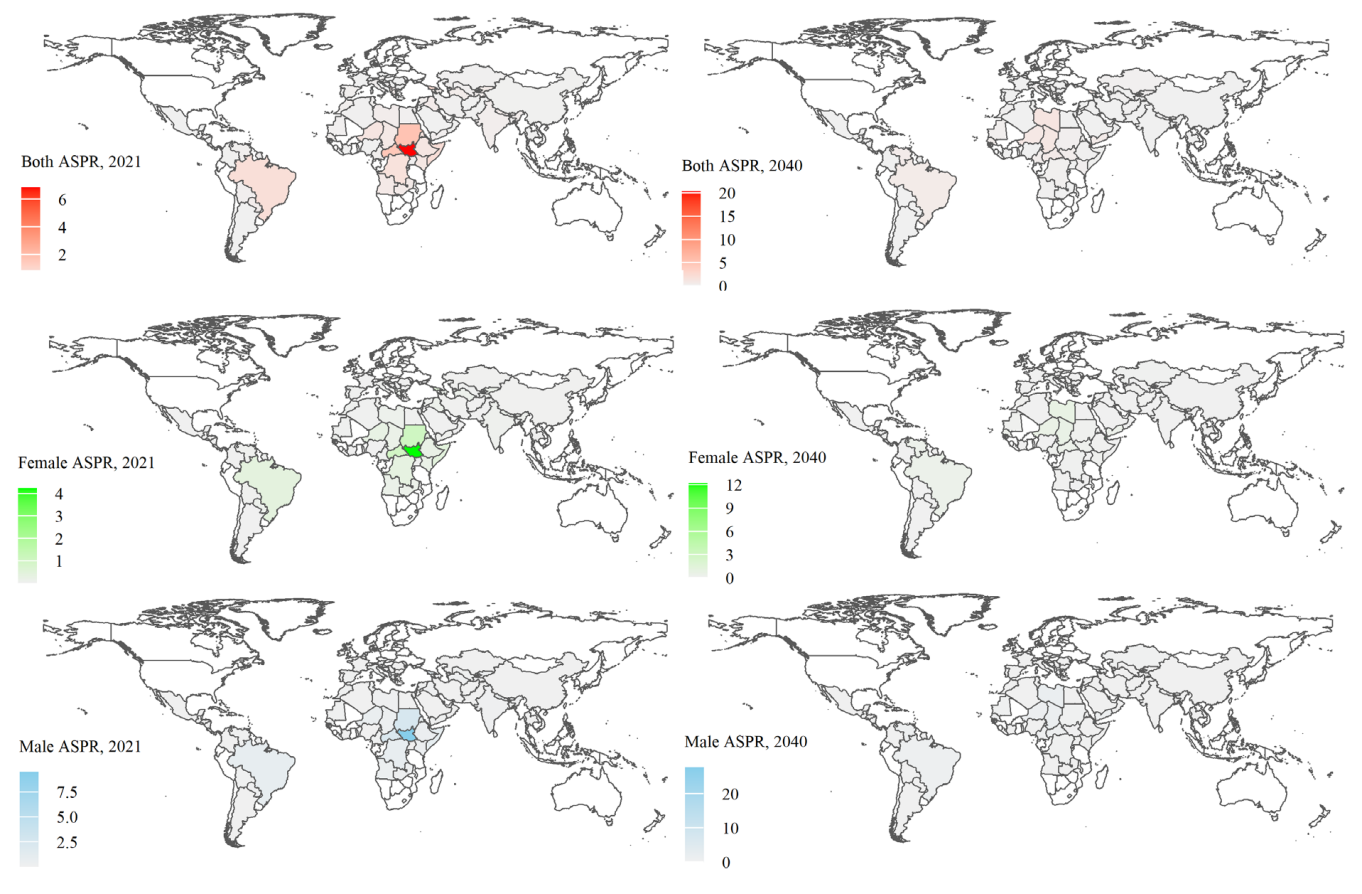
ASPR per 100,000 of VL for both sexes and for females and males separately in endemic countries in 2021 and 2040. Note. ASPR: Age-standardized prevalence rates; VL: Visceral leishmaniasis. In each case, the white-colored polygons represent disease-free nations

 Among nations, Djibouti is anticipated to exhibit the greatest ASPR by 2040, reaching 20.32 (95% CI: 5.25–78.67), reflecting an extraordinary increase of 2050.59% from 2021 to 2040. Following Djibouti, Libya, and Chad are projected to record the highest ASPR values of 1.28 and 1.10, respectively, with increases of 760.05% and 288.11%. In contrast, Romania and Nigeria are expected to have the lowest ASPR rates, at 0.00016 and 0.00020, respectively, with substantial decreases of -97.19% and -95.91% by 2040. Moreover, among 83 endemic countries, only 27 countries are projected to experience an increase in ASPR. Specifically, Djibouti (2050.59%), Venezuela (818.24%), Yemen (763.52%), and El Salvador (661.72%) will experience the highest increases in ASPR. Conversely, Spain (-98.86%), Bangladesh (-96.89%), and South Sudan (-95.66%) are anticipated to exhibit marked declines by 2040. This variability underscores the heterogeneous epidemiological patterns of VL across nations (Figure 3).

###  Sex-based national prevalence of visceral leishmaniasis 

 Among females, Djibouti is expected to have the highest ASPR by 2040, at 12.0, reflecting a significant increase of 2058.44% from 2019 to 2040. Libya and Chad follow with ASPR values of 0.78 and 0.66, respectively, corresponding to increases of 764.05% and 407.20%. In contrast, Spain, Nigeria, and Romania are projected to have the lowest ASPR, at 0.00033, 0.00013, and 0.00010, respectively, with declines of -98.85%, -96.91%, and -97.17% by 2040. From 1990 to 2021, Niger (481.20%), Chad (407.20%), and Uzbekistan (364.49%) recorded the largest increases in ASPR, whereas Pakistan (-99.95%), Afghanistan (-99.84%), and Romania (-99.37%) exhibited the greatest declines. Furthermore, from 2021 to 2040, the highest increases in ASPR are expected in Djibouti (2058.43%), Venezuela (839.42%), and Libya (764.05%), while Spain (-98.84%), Romania (-97.16%), and Bangladesh (-96.90%) are projected to show the sharpest decreases. For males, Djibouti is projected to have the highest ASPR at 27.65 by 2040, reflecting a dramatic increase of 2060.38% from 2019 to 2040. Libya and Chad follow with ASPR values of 1.79 and 1.56, respectively, showing increases of 787.15% and 409.73% from 2021 to 2040. Conversely, Spain, Nigeria, and Romania are anticipated to have the lowest ASPR among males, at 0.00076, 0.00029, and 0.00021, with declines of -98.85%, -96.92%, and -97.42% from 2019 to 2040 2040. Overall, these findings suggest that males generally experience higher ASPR values compared to females (Figure 3). However, the percentage changes in ASPR from 2021 to 2040 remain broadly comparable between the two sexes in most countries.

###  Decomposition analysis

**Figure 4:**
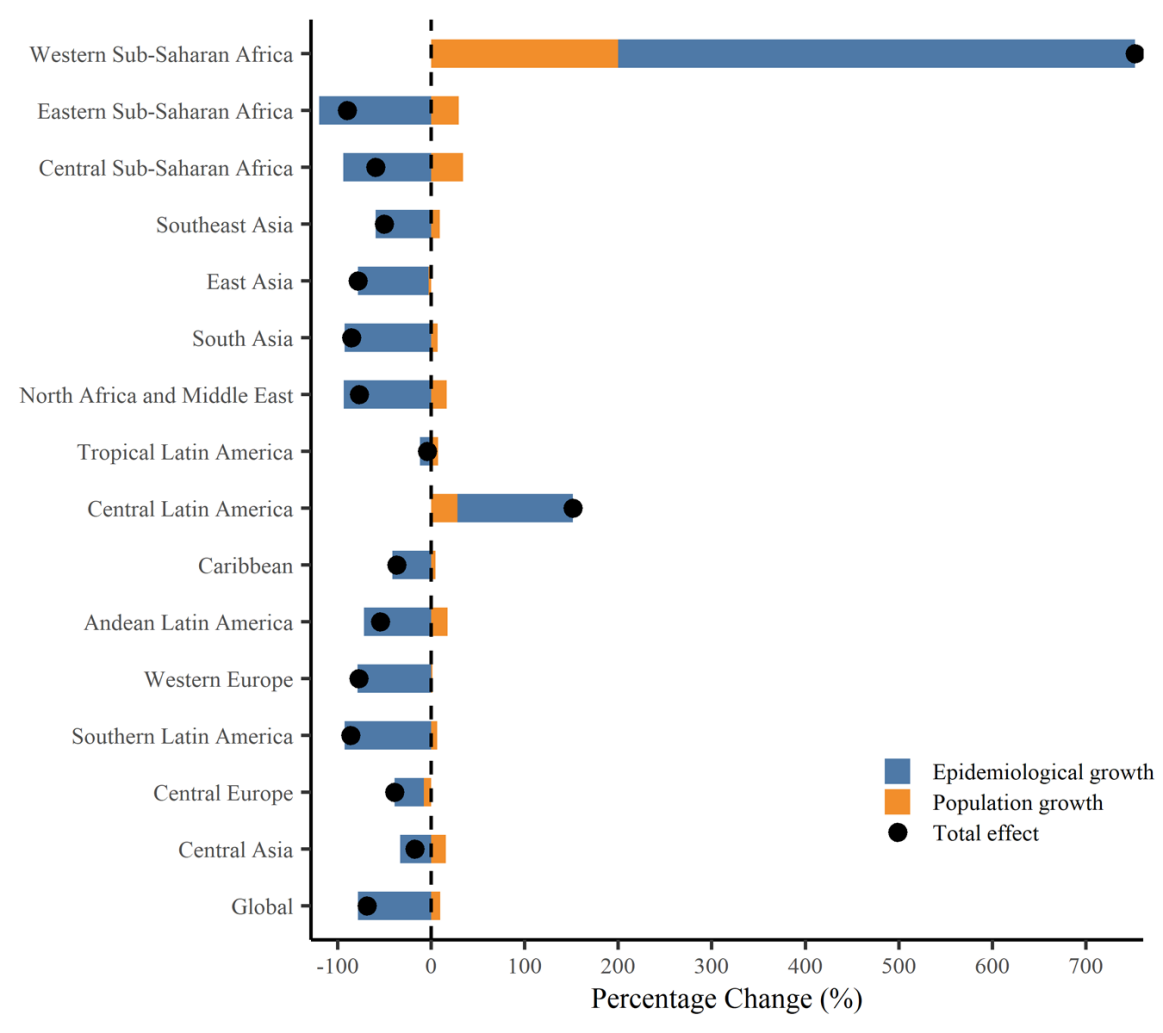
Das Gupta decomposition of the age-standardized prevalence of VL during 2021–2040, Globally and Across 15 Endemic Regions for Both Sexes. Note. VL: Visceral leishmaniasis

 The Das Gupta decomposition analysis reveals that the projected change in the ASPR by 2040 is primarily determined by shifts in epidemiological rates, with demographic changes serving as a secondary but influential factor. Globally, the projected 68.4% decrease in ASPR is largely attributable to a substantial epidemiological effect (-78.1%), which is partially offset by counteracting population growth ( + 9.7%). This dominance of the epidemiological pattern is consistent across most regions. Notable exceptions highlight distinct underlying drivers. For instance, Western Sub-Saharan Africa is projected to experience a dramatic 752% increase in ASPR, resulting from a powerful synergistic combination of both rapid population growth ( + 200%) and a sharply worsening epidemiological rate ( + 552%). In contrast, the 152% increase in Central Latin America reflects a more balanced contribution from both factors (Figure 4).

## Discussion

 Our study provides critical insights into the evolving burden of VL from 1990 to 2040, contributing to global efforts to reduce its prevalence while informing healthcare system planning at global, regional, and national levels. Globally, the ASPR of VL remarkably declined by -94.68% from 1990 to 2021, with projections indicating a further reduction of -72.55% by 2040. These trends align with global health strategies, including the WHO’s kala-azar elimination program, which has significantly reduced VL prevalence in high-burden regions such as South Asia (-97.98% from 1990 to 2021) through improvements in vector control, diagnostics, and treatment access.^21,22^ However, substantial regional and national variations persist, with some areas experiencing alarming increases that highlight the need for more targeted interventions.

 Regionally, Tropical Latin America continues to have the highest projected ASPR, estimated at 0.583 (95% CI: 0.565–0.602) per 100,000 by 2040, despite a modest decline of -11.35%. This persistence may be attributed to environmental factors favorable to sandfly vectors and ongoing challenges in sustaining effective control programs in rural and remote areas.^15^ Conversely, Western Sub-Saharan Africa and Central Latin America are projected to experience dramatic increases in ASPR (417.12% and 113.34%, respectively), potentially driven by climate change, population displacement, and limited healthcare infrastructure.^23,24^ These projections differ markedly from those of Southeast Asia and the Caribbean, which exhibit the lowest ASPR (0.00013 and 0.00033 per 100,000, respectively), reflecting effective VL control measures and low vector prevalence. The historical increase in ASPR in Western Sub-Saharan Africa (269.17% from 1990 to 2021) further underscores the region’s vulnerability, possibly due to inadequate surveillance and resource constraints.^25^

 These divergent regional patterns are primarily attributed to epidemiological factors and subsequent population changes. Therefore, further epidemiological studies are essential to compare these regions and clarify the underlying causes of these differences. Such research will be instrumental in accelerating global efforts to effectively combat VL.

 Sex-based analyses reveal that males generally exhibit higher ASPR values than females, particularly in Tropical Latin America, where the projected 2040 rates are 0.811 for males compared to 0.358 for females. This disparity may stem from sex-specific exposure patterns, such as greater occupational exposure among males and increased involvement in outdoor activities that elevate their risk of contact with sandfly vectors. In contrast, indoor activities, often undertaken by females, may result in reduced exposure to these vectors. Furthermore, disparities in healthcare access can exacerbate these differences, as males in certain settings are less likely to seek timely medical attention or engage in preventive measures. Biological factors, including sex-related variations in immune responses, may further contribute to the observed prevalence rates. Understanding these multifaceted factors is essential for developing targeted public health strategies to reduce VL incidence and addressing the specific needs of diverse demographic groups.^26-28^

 Nationally, Djibouti, Libya, and Chad are projected to have the highest ASPR of VL by 2040, with Djibouti experiencing an alarming 2050.59% increase, which raises significant public health concerns. Similar increases are also expected in Venezuela and Yemen, reflecting the damaging effects of political instability and weakened health systems that impair vector control measures and restrict access to treatment, thereby exacerbating disease transmission.^29^ Additionally, these patterns are shaped by ecological factors such as desertification, which alters sandfly habitats and boosts human exposure. Socioeconomic challenges, including conflict and population displacement, significantly impact these situations by disrupting healthcare infrastructure and creating overcrowded environments that enable rapid disease spread.^26,30,31^ Addressing these complex and interrelated factors calls for more comprehensive epidemiological studies examining how environmental, social, and health-system factors intersect to influence VL dynamics.

 In contrast, countries such as Romania, Nigeria, and Spain exhibit negligible ASPR values accompanied by substantial declines (-97.19%, -95.91%, and -98.86%, respectively), likely due to robust public health systems and low sandfly prevalence.^32^ The decline observed in Romania, for instance, mirrors trends seen in other high-income countries where strong surveillance and environmental management have significantly reduced transmission.^33^ However, the rapid projected increases in certain nations underscore the persistent challenges of maintaining VL control in resource-limited settings.

 Moreover, the substantial reduction in VL prevalence in regions such as South Asia can be attributed to integrated control programs, including the use of insecticide-treated nets, indoor residual spraying, and early diagnosis by rapid diagnostic tests.^34^ However, the rising trends observed in Western Sub-Saharan Africa and several high-burden nations highlight systemic challenges, including limited access to healthcare, high costs of diagnostics and treatment, and chronic underreporting in low-resource settings.^35^ In Sub-Saharan Africa, where two-thirds of the world’s impoverished population resides, the absence of comprehensive health insurance systems further exacerbates these challenges, restricting access to affordable care.^36^ Moreover, inadequate data collection and surveillance systems in these regions may underestimate true VL prevalence, underscoring the need for improved data quality to enhance the reliability of predictive models.^37^

 This study has several strengths, including its comprehensive global, regional, and sex-specific projections of VL prevalence, supported by robust statistical methods such as the enhanced IDM and Das Gupta decomposition analysis. These approaches allow for a detailed assessment of VL trends across 98 endemic countries and regions, thereby facilitating more targeted public health interventions.

 However, this study has several limitations. Model parameters were calibrated using historical data, and the lack of an independent dataset precluded external validation. Although a formal sensitivity analysis was not conducted, uncertainty was quantified through confidence intervals derived from the calibration process. Furthermore, the model structure itself accounted for interactions among key epidemiological parameters (e.g., incidence, remission, and mortality), which contributes to internal robustness. Furthermore, this study relied on historical data from the GBD (1990–2021), which may not fully capture unforeseen influences such as climate change, political instability, or therapeutic advancements.^23,26,38^ Underreporting poses a significant challenge, particularly in low-income or conflict-affected regions such as Western Sub-Saharan Africa, Djibouti, Yemen, and Chad.^37,39^ This underreporting likely leads to underestimations of true incidence and prevalence, potentially biasing ASPR projections in regions with weak surveillance systems. These data deficiencies help explain the wide confidence intervals observed in some projections, such as the notable increase forecast for Djibouti, where limited and volatile historical data provide less information for model calibration, resulting in a broader range of plausible future outcomes.

 Therefore, while these projections highlight areas at potentially high risk, the precise magnitude of these increases remains uncertain. These estimates should be interpreted as indicators warranting enhanced surveillance rather than precise forecasts.

 To address these limitations, future research should incorporate real-time surveillance data to improve data quality and reduce underreporting biases.^37^ Community-based reporting systems and cross-border surveillance initiatives, such as those supported by the African Union,^25^ could enhance data accuracy. Additionally, modeling the impact of environmental factors (e.g., climate-driven changes in sandfly habitats), socio-economic conditions (e.g., population displacement), and technological advancements (e.g., new diagnostics or vaccines) would help refine projections and address current gaps.^23,38^ In conclusion, while global declines in VL prevalence are encouraging, the projected increases in specific regions and countries, particularly Western Sub-Saharan Africa and Djibouti, underscore the need for urgent, targeted interventions. Strengthening healthcare systems, enhancing vector control efforts, and addressing sex-specific risk factors will be critical to sustaining progress and mitigating emerging public health challenges.

**Highlights**

- Global visceral leishmaniasis (VL) prevalence is projected to continue declining through 2040.
- Western Sub-Saharan Africa is projected to experience a five-fold increase in ASPR.
- Epidemiological factors are the primary driver of future VL ASPR changes.
- Enhanced surveillance in high-risk regions is crucial for sustaining disease control.
- Only 27 of 83 endemic countries are expected to show an increasing ASPR.

## Conclusion

 This study provides a comprehensive outlook on VL prevalence trends through 2040 using an enhanced illness-death modeling framework. The substantial reduction in global ASPR by 2040 underscores the continued effectiveness of initiatives such as the WHO kala-azar elimination program, which prioritizes improved vector control, early diagnosis, and expanded access to treatment. However, notable regional and national variations remain. Tropical Latin America is projected to maintain the highest disease burden, while Western Sub-Saharan Africa and countries such as Djibouti, Libya, and Chad face steep ASPR increases, likely driven by environmental factors such as climate change, population displacement, and weak healthcare infrastructure. Sex disparities, including higher ASPR in males across most regions, highlight the need for sex-tailored interventions. To sustain global progress and address projected increases, continued investment in robust surveillance systems, strengthened vector control programs, and equitable access to diagnostic and treatment services is essential. Future research should integrate real-time surveillance data and incorporate environmental and socio-economic factors into predictive models to refine predictions and guide more effective interventions.

## Acknowledgements

 The authors acknowledge the use of DeepSeek, an AI-based tool, for editing and improving the manuscript to enhance clarity and coherence. All modifications were reviewed and approved by the authors.

## Competing Interests

 The authors have no conflict of interests.

## Ethical Approval

 This project has been approved by the Ethics Committee at Qom University of Medical Sciences, Qom, Iran. (NO: IR.MUQ.REC.1403.287).

## Funding

 This study was funded by Qom University of Medical Sciences (Grant #3745).
